# Diagnostic Performance of Serum CK-MB, TNF-α and Hs-CRP in Children with Viral Myocarditis

**DOI:** 10.1515/biol-2019-0005

**Published:** 2019-03-20

**Authors:** Jia Chen, Yuanying Deng

**Affiliations:** 1 No.138 Xintongpo Road Hexi Yuelu District Changsha City Hunan Province 410013 PR China; 2 Department of Pediatrics, The Third Xiangya Hospital of Central South University 410013, Changsha China

**Keywords:** CK-MB, TNF-α, hs-CRP, viral myocarditis

## Abstract

**Objective:**

The purpose of this study was to investigate the diagnostic performance of serum CK-MB, TNF-α and hs-CRP in children with viral myocarditis (VMC).

**Methods:**

Fifty-six children with confirmed diagnosis of VMC were included in this study. Of the included 56 cases, 25 subjects were in acute and other 31 were in the recovery stage. A contemporaneous control group of 22 children were included for comparison. The serum concentration of CK-MB, TNF-α and hs-CRP were examined in both VMC and control groups.

**Results:**

The serum concentration of CK-MB, TNF-α and hs-CRP were 31.77±9.48 (UL), 143.11±23.27 (ng/L) and 8.10±1.94(mg/L) for acute stage VMC; 12.72±4.99 (UL), 83.15±13.35 (ng/L) and 4.07±1.12 (mg/L) for recovery stage VMC; 8.11±3.20 (UL), 68.27±12.55 (ng/L) and 2.56±1.27 (mg/L) for control group respectively; The serum concentration of CK-MB, TNF-α and hs-CRP were significantly different between acute stage VMC, recovery stage VMC and control groups (p<0.05); Significant positive correlation between CK-MB and hs-CRP were found in acute stage VMC (r=0.54, p=0.01) and recovery stage VMC (r=0.37, p=0.04). Using serum CK-MB, TNF-α and hs-CRP as the reference, the differential diagnosis sensitivity for acute and recovery stage VMC were 87.10 (70.17-96.37)%, 87.10 (70.17-96.37)% and 77.42 (58.90-90.415)%; The specificity were 92.00 (73.97-99.02)%, 96.00 (79.65-99.90)% and 100.00 (86.28-100.00)% respectively.

**Conclusion:**

Serum concentration of CK-MB, TNF-α and hs-CRP in children with VMC were significant increased especially in acute stage, which can be used as biomarkers for VMC diagnosis.

## 1 Introduction

Viral myocarditis (VMC) is an infectious cardiomyopathy that refers to myocardial limitation, diffuse acute, or chronic inflammatory lesions caused by viral infections [1]. Epidemiological studies have shown that different viruses can cause myocarditis, and the most common viral cause an also result in intestinal and upper respiratory tract infections[2, 3]. Coxsackie virus group A, Coxsackie virus group B, ECHO virus, and poliovirus are common myocarditis-causing viruses [4]. Among them, Coxsackie virus group B is the most commonly identified in VMC patients. Other viruses, such as adenovirus, influenza, and parainfluenza viruses, can also lead to viral myocarditis [5]. Children are susceptible to viral myocardial infections, which are common cardiovascular diseases in children. VMC in children is clinically diagnosed, but specific diagnostic indicators are unavailable. As such, mastering and implementing them are difficult. Increased levels of certain inflammatory factors in the peripheral blood of children with VMC can be used for the auxiliary diagnosis of VMC [6, 7]. In this study, we investigated the serum levels of CK-MB, TNF-α, and hs-CRP [8], which were used as serum markers, to identify the VMC values in acute and recovery phases in children with acute and convalescent VMC and in non-infected children.

## 2 Material and methods

### 2.1 Patient inclusion criteria

Fifty-six children with VMC were admitted to our hospital from March 2015 to February 2017 and selected as study subjects. The following inclusion criteria were considered: children who were aged 2 to 12 years, children who satisfied the PVM diagnostic criteria established by the Chinese Medical Association, children who provided a signed informed consent, and children who voluntarily joined the study. The following exclusion criteria were: children with unclear VMC diagnosis, congenital heart disease, severe pulmonary infection, congenital immunodeficiency, severe hepatic and renal insufficiency, and relevant drug allergies. Twenty-two normal children in our hospital during the same period were selected as the control group. In the VMC group, 25 children, including 14 males and 11 females aged 3–12 years, were at the acute stage (within 7 days of onset). All the patients had a history of upper respiratory tract infection within 3 weeks before onset. Of these children, 21 were positive for coxB IgM, and 4 were positive for anti-adenovirus IgM. Furthermore, 31 cases, including 18 cases of males and 13 cases of females aged 2–12 years, were in the recovery period (2–3 months after onset). The control group consisted of 22 cases, including 14 males and 8 females aged 2–14 years.

**Informed consent**: Informed consent has been obtained from all individuals included in this study

**Ethical approval**: The research related to human use has been complied with all the relevant national regulations, institutional policies and in accordance the tenets of the Helsinki Declaration, and has been approved by the authors’ institutional review board or equivalent committee.

### 2.2 Serum CK-MB, TNF-α and hs-CRP examination

Venous blood (5 ml) was taken from the children in the VMC group early in the morning on their 2nd day of hospital admission. Venous blood (5 ml) was also drawn from the children in the normal control group early in the morning on the day of physical examination [9]. Serum was routinely separated to determine TNF-α, hs-CRP, CK-MN, and other indicators. Serum TNF-α levels were measured through ELISA, and serum hs-CRP levels were determined through immunoturbidimetry assay. CK-MB was used to determine, and measurements were performed with a Hitachi 7180 type automatic biochemical analyzer by using a McKay reagent. TNF-α and hs-CRP reagents were provided by Shanghai Shenneng Desai Biotechnology Co., Ltd.

### 2.3 Statistical analysis

Data was expressed as the mean ± sd and analyzed by Spss17.0 software (SPSS, Inc., Chicago, IL, USA). The difference between acute and recovery group were assessed by Student’s-t test. Diagnostic sensitivity and specificity was calculated by the equation of sensitivity=true positive/(true positive+ false negative), specificity=true negative/( true negative+ false positive). The area under the receiver operating characteristic (ROC) curve was used to evaluate the feasibility of serum CK-MB, TNF-α and hs-CRP as biomarkers for acute and recovery VMC differential diagnosis. Two tailed p<0.05 was considered statistically significant.

## 3 Results

### 3.1 Serum concentration of CK-MB, TNF-α and hs-CRP

**Figure 1:**
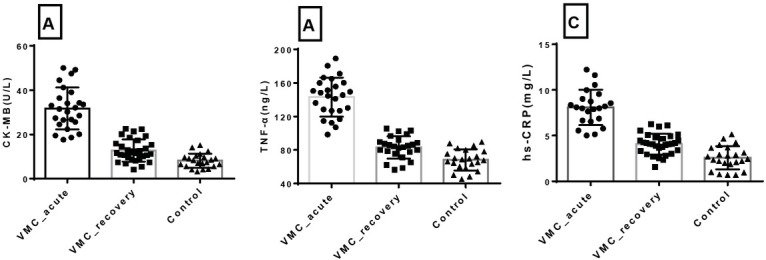
Scatter plot of serum concentration of CK-MB, TNF-α and hs-CRP in different groups (A:Serum CK-MB; B:Serum TNF-α; C:Serum hs-CRP)

The serum concentration of CK-MB, TNF-α and hs-CRP were 31.77±9.48 (UL), 143.11±23.27 (ng/L) and 8.10±1.94(mg/L) for acute stage VMC; 12.72±4.99 (UL), 83.15±13.35 (ng/L) and 4.07±1.12 (mg/L) for recovery stage VMC; 8.11±3.20 (UL), 68.27±12.55 (ng/L) and 2.56±1.27 (mg/L) for control group respectively (Table 1); The serum concentration of CK-MB, TNF-α and hs-CRP were statistical different for acute stage VMC, recovery stage VMC and control group (p<0.05), Figure 1.

**Table 1:** Serum concentration of CK-MB, TNF-α and hs-CRP comparison in VMC and control groups

Group	n	CK-MB(U/L)	TNF-α(ng/L)	hs-CRP(mg/L)
VMC				
Acute stage	25	31.77±9.48	143.11±23.27	8.10±1.94
Recovery stage	31	12.72±4.99	83.15±13.35	4.07±1.12
Control	22	8.11±3.20	68.27±12.55	2.56±1.27
F value		93.15	126.90	88.55
P value		<0.0001	<0.0001	<0.0001

### 3.2 Serum CK-MB, TNF-α and hs-CRP correlation

**Figure 2:**
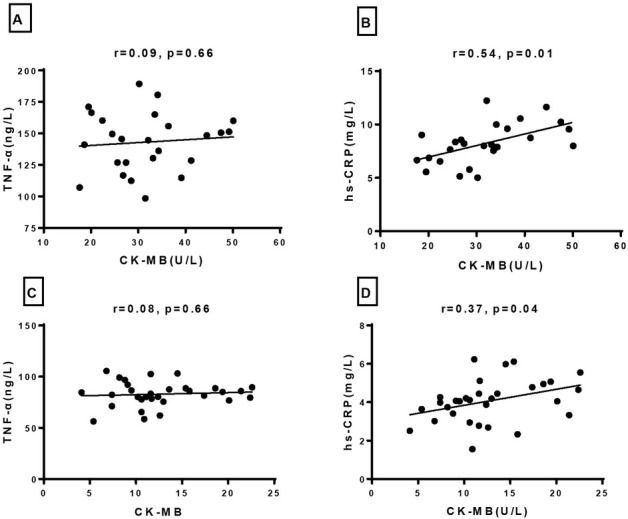
Pearson correlation analysis for serum CK-MB and TNF-α, hs-CRP in acute and recovery stage VMC (A: Correlation between CK-MB and TNF-α in acute stage VMC; B: Correlation between CK-MB and hs-CRP in acute stage VMC; C: Correlation between CK-MB and TNF-α in recovery stage VMC; D: Correlation between CK-MB and hs-CRP in recovery stage VMC)

Correlation between CK-MB and TNF-α, hs-CRP were analyzed by Pearson correlation test. Significant positive correlation between CK-MB and hs-CRP were found in acute stage VMC (r=0.54, p=0.01) and recovery stage VMC(r=0.37, p=0.04); However, no correlation was found between CK-MB and TNF-α in both acute and recovery stage VMC, Figure 2.

### 3.3 CK-MB, TNF-α and hs-CRP as biomarker for acute and recovery VMC differential diagnosis

Using serum CK-MB, TNF-α and hs-CRP as the reference, the differential diagnosis sensitivity for acute and recovery stage VMC were 87.10 (70.17-96.37)%, 87.10 (70.17-96.37)% and 77.42 (58.90-90.415)%; The specificity were 92.00 (73.97-99.02)%, 96.00 (79.65-99.90)% and 100.00 (86.28-100.00)% respectively, Table 2.

**Table 2:** Diagnostic performance of serum CK-MB, TNF-α and hs-CRP correlation

Reference	Sen (95%CI)	Sep(95%CI)	AUC(95%CI)	Cut off
CK-MB(U/L)	87.10(70.17-96.37)%	92.00(73.97-99.02)%	0.97(0.94-1.00)	19.45
TNF-α(ng/L)	87.10(70.17-96.37)%	96.00(79.65-99.90)%	0.99(0.98-1.00)	98.86
hs-CRP(mg/L)	77.42(58.90-90.415)%	100.00(86.28-100.00)%	0.98(0.95-1.00)	4.98

## 4 Discussion

VMC is common in clinical practice, and is mainly caused by Coxsackie virus, Ekage virus, and adenovirus 40 and 41 [10]. These viruses often directly damage cardiomyocytes in children and release relevant antigens to stimulate an immune response, which in turn disrupts the immunity of cardiomyocytes [1, 11, 12]. Affected children often show fever, body aches, sore throat, and other flu-like symptoms, such as flustered feelings and shortness of breath, and may suffer from myocardial damage and changes in ECG[13]. The diagnostic criteria of VMC in China have been revised several times. In 1994, the current diagnostic criteria were established. However, the current VMC diagnostic criteria still have some problems [14, 15]. (1) The diagnostic criteria are complex, and clinical practice is difficult. (2) The diagnostic criterias are broad, and high rates of misdiagnosis may occur. (3) The symptom index of a child is unclear, physical index lacks specificity, and aspartate aminotransferase levels may increase in other diseases, which are the main reasons for the expansion of diagnosis. (4) Viruses or virus antibodies isolated from myocardial or blood specimens have the same value in terms of the diagnosis of viral pathogens, but obtaining myocardial specimens is difficult. Therefore, accurate, convenient, and clinically viable VMC diagnostic methods should be explored.

The serum levels of TNF-α [16] and hs-CRP in children with VMC decreased significantly and gradually improved as the disease progressed compared with those in healthy children. The dynamic changes in these inflammatory factors reflect the development of children with VMC, and they can be used as serological markers for the diagnosis of VMC and the differential diagnosis of acute and convalescent VMC.

TNF-α is a cytokine secreted by monocytes and macrophages. It can kill or inhibit tumor cells and enhance neutrophil phagocytosis. TNF-α has biological activities, such as inducing anti-infection effectsand promoting cell proliferation and differentiation, which can be directly involved in immune processes caused by myocarditis and other diseases [17]. Serum TNF-α and mRNA expression in patients with viral myocarditis increase. TNF-α is involved in the pathogenesis of viral myocarditis, and TNF-α antibody treatment can significantly reduce the degree of myocardial damage and mortality. In this study, serum TNF-α in children with recurrent VMC was significantly higher than that in normal children, and the acute phase group was higher than the recovery phase group.These observations were consistent with the above results. Serum TNF-α could be used as a serological marker to evaluate VMC severity and progression.

CRP is a typical acute-phase reaction protein that abnormally increases under stress conditions, such as infection and tissue damage. It is synthesized by hepatocytes and exists in the form of glycoproteins, which can activate complement and enhance the ability of phagocytic cells to engulf pathogenic microorganisms and necrotic and apoptotic cells. Serum CRP level increases abnormally at the early stage of myocardial injury[9]. Our study found that the serum hs-CRP level in children with VMC was positively correlated with the CK-MB level, suggesting that it could be used as a serological indicator to assess the degree of myocardial damage in children with VMC. However, we only included 56 subjects in the present work. The statistical power is limited with small sample sizes. Therefore, a prospective clinical study using larger sample sizes or high quality meta-analysis were needed to further evaluate the diagnostic performance of serum CK-MB, TNF-α and hs-CRP in children with viral myocarditis.

In summary, serum TNF-α and hs-CRP were significantly increased in children with acute VMC, and serum TNF-α and hs-CRP levels gradually decreased as the disease progressed. Serum TNF-α and hs-CRP could be used as serological markers to identify VMC in acute and recovery phases.

## References

[1] Uhl TL (2008). Viral myocarditis in children. Crit Care Nurse.

[2] Robinson J, Hartling L, Vandermeer B, Crumley E, Klassen TP (2005). Intravenous immunoglobulin for presumed viral myocarditis in children and adults. Cochrane Database Syst Rev.

[3] Lv S, Rong J, Ren S, Wu M, Li M, Zhu Y, Zhang J (2013). Epidemiology and diagnosis of viral myocarditis. Hellenic J Cardiol.

[4] Niu L, An XJ, Tian J, Wang Y (2015). 124 cases of clinical analysis of children with viral myocarditis. Eur Rev Med Pharmacol Sci.

[5] Talmon G, Fink DL, Horowitz Y, Miron D (2015). [THE PREVALENCE OF SUBCLINICAL MYOCARDITIS AMONG YOUNG CHILDREN WITH ACUTE VIRAL INFECTION]. Harefuah.

[6] Corsten MF, Schroen B, Heymans S (2012). Inflammation in viral myocarditis: friend or foe. Trends Mol Med.

[7] Heymans S (2006). Inflammation and cardiac remodeling during viral myocarditis. Ernst Schering Res Found Workshop.

[8] Wang D, Li T, Cui H, Zhang Y (2016). Analysis of the Indicating Value of Cardiac Troponin I, Tumor Necrosis Factor-α, Interleukin-18, Mir-1 and Mir-146b for Viral Myocarditis among Children. Cell Physiol Biochem.

[9] Guo JG (2008). [Detection of cardiac troponin and high-sensitivity C reactive protein in children with viral myocarditis]. Nan Fang Yi Ke Da Xue Xue Bao.

[10] Suddaby EC (1996). Viral myocarditis in children. Crit Care Nurse.

[11] Al-Biltagi M, Issa M, Hagar HA, Abdel-Hafez M, Aziz NA (2010). Circulating cardiac troponins levels and cardiac dysfunction in children with acute and fulminant viral myocarditis. Acta Paediatr.

[12] Dennert R, Crijns HJ, Heymans S (2008). Acute viral myocarditis. Eur Heart J.

[13] Frey T, Arain N (2018). Pediatric Viral Myocarditis - A Review. S D Med.

[14] Pollack A, Kontorovich AR, Fuster V, Dec GW (2015). Viral myocarditis-diagnosis, treatment options, and current controversies. Nat Rev Cardiol.

[15] Rose NR (2016). Viral myocarditis. Curr Opin Rheumatol.

[16] Calabrese F, Carturan E, Chimenti C, Pieroni M, Agostini C, Angelini A, Crosato M, Valente M, Boffa GM, Frustaci A, Thiene G (2004). Overexpression of tumor necrosis factor (TNF)alpha and TNFalpha receptor I in human viral myocarditis: clinicopathologic correlations. Mod Pathol.

[17] Zhang S, Rao B (2002). The expression of TNF-alpha mRNA and its significance in murine viral myocarditis. Zhonghua Bing Li Xue Za Zhi.

