# Erratum to “Diagnostic performance of serum CK-MB, TNF-α and hs-CRP in children with viral myocarditis”

**DOI:** 10.1515/biol-2020-0073

**Published:** 2020-08-18

**Authors:** Jia Chen, Yuanying Deng

In the published article “Chen J, Deng Y. Diagnostic performance of serum CK-MB, TNF-α and hs-CRP in children with viral myocarditis. *Open Life Sci.* 2019;14:38–42. doi: 10.1515/biol-2019-0005” the affiliation of author Jia Chen is incorrect.

The correct affiliation of Jia Chen is as follows: Department of Pediatrics, The Third Xiangya Hospital of Central South University, No. 138 Tongzipo Road Hexi Yuelu District, Changsha City, Hunan Province 410013, P.R. China.

